# Supplementary material for: Chromothripsis during telomere crisis is independent of NHEJ, and consistent with a replicative origin
Source: Genome Res. 2019 May;29(5):737–49. doi: 10.1101/gr.240705.118 (PMC6499312; doi:10.1101/gr.240705.118)
Supplement: Supplemental Material [file supp_gr.240705.118_Supplemental_file_1.zip › contigs/annotated_contigs/DB84/contig.5.DB84_length_1150_mean_cov_8.42347826087.docx]

**DB84_length_1150_mean_cov_8.42347826087**

```
AGCCAGAGATTCCTATCGGGAGAGGGAAGACTTTACTTTTGAAGAGGAAAAGTACTTAACTCCTAGAGGAAAGCGTCATTCCTGAGGTT
 >chr17:8573898-8574282 + E=7e-219 p=1e-02
TACCCTCATGGTCTGGAGACAAACACGTGGGACTTTTCTTTTTTGTGTGTGTTTCTAATAGTGGAAGAAAAGGAAAGCCAGGCTAAGCA

GAGAAAAGTGGAAGAGGAGACACAGCAGCTCATTCTTTCCCAGTAGCCGTTAGACTGATTGCGGAGGGGAAGGTCAAAAGAGAAGTTCA

CAACTATGATACCAGCTCTAGAGGCGAAAGAACAACTGAGAGAGAACTATCCAAGAAGTGTTTGGATCCCGAAGCACAAAGGTGAGGAA

AGAGCAAAATATGGCTTTCCCTCATAAA|TAATAATTATTATT|ATTTTAATAATAATAAAAAATAAGGATTATTAAATGTGTTGTAAA
                                           >chr8:83541460-83541732 - E=1e-148 p=1e-02
AGATAGCAGTTACATGAAATTAACTTAATTTTCTCTTTTATTAAAAAAAGGAAATTAACAGTTAGTTGGAAAATTAAACCACTGAAGTT

ATTTAGCTATTATTTTCTCTATGAACTTTTCATGATACAAATTGCATATATTTGATTAAAATAAACAAAAAATGGAATTTCTGCACATG

TATTCCAGGACTTTAAATAAAATAAAATATTTAAAAATAAAAATAATA|A|TATATATATATTATATATATATATAATATATATATATA
                                                 >chr2:108340900-108340949 - E=2e-14
TATATATATA|AGT|AAGCCAAAGGCCGGGTCCGGCGGCTCATGCCTATAATCCCAGCACTTTGGGAGGCTGAAGCAGGCGGATCATCT
              >chr10:18464694-18464834 - E=7e-23 p=0e+00
GAGGTCGGGAGTTCGAGACCAGCCCTGACCAACATAGAGAAACCCCATCTCTACTAAAAATACAAAA|TTAG|CCAGGCGTGGTGGCAC
                                                                        >chr7:99146379-
ATGGCCTGTAATTCCAGCTACTTGGGAGGCAGAGGCAGGAGAATCACTTTAACCTGGGAAGTGGAGGTTGCAGTGAGCCGAGATCGTGC
99146666 - E=2e-153
CCTTGCACTCCAGCCTGGGCAACAAGAGCGAAACTCCATCTCAAAAAAAAAAAAAAAAAGAATGACCAGATCACTTTTTAGAGTTGGCA

GTGTAGCAGCATATTTAGGACACTCGCTTCAGACTTGGATTTGAGTGCTGGTTCTATACTTACTGTGGGTTGTTAGACACATTTAATCT

T
```
